# Ultrasound-guided acupotomy for trigger finger: a systematic review and meta-analysis

**DOI:** 10.1186/s13018-023-04127-3

**Published:** 2023-09-13

**Authors:** Yong-shan Liang, Ling-yan Chen, Yao-yun Cui, Chun-xiao Du, Yun-xiang Xu, Lun-hui Yin

**Affiliations:** 1 Clinical Medical School of Acupuncture, Moxibustion and Rehabilitation, Guangzhou University of Chinese Medicine, Guangzhou, 510405 Guangdong China; 2 Department of Rehabilitation, The First Affiliated Hospital of Guangzhou Medical University, Guangzhou, 510120 Guangdong China; 3 Guangdong Lingnan Institute of Technology, Guangzhou, 510640 Guangdong China

**Keywords:** Trigger finger, Ultrasound, Acupotomy, Efficacy, Safety, Meta-analysis

## Abstract

**Background:**

Trigger finger is a common condition in the hand, and ultrasound-guided acupotomy for trigger finger has been widely used in recent years.

**Purpose:**

This study aims to investigate the efficacy and safety of ultrasound-guided acupotomy for trigger finger.

**Methods:**

We searched for relevant studies in the Cochrane Library, China National Knowledge Infrastructure (CNKI), Embase, PubMed, Chinese Biomedical Literature Database (CBM), Wanfang Data, and other resources from their inception to January 2023. Randomized controlled trials of ultrasound-guided acupotomy for trigger finger were included. The meta-analysis was carried out using Review Manager 5.4 and Stata 15.1.

**Results:**

Overall, 15 studies with 988 patients were included. The experimental group was treated with ultrasound-guided acupotomy, and the Control group received traditional acupotomy, traditional operation or injection of medication. Meta-analysis showed that the overall clinical effectiveness (OR = 4.83; 95% CI 2.49–9.37; *I*^2^ = 73.1%; *P* < 0.001) in the experimental group was significantly better than that of the control group. And the Visual Analogue Scale (VAS) score (WMD =  − 1; 95% CI − 1.24, − 0.76; *I*^2^ = 99%; *P* < 0.001), the QuinneII classification (WMD = − 0.84; 95% CI − 1.28, − 0.39; *I*^2^ = 99.1%, *P* < 0.001), the incidence of complications (RR = 0.26; 95% CI 0.11, 0.63; *I*^2^ = 0%, *P* = 0.003), and the recurrence rate (RR = 0.14; 95% CI 0.03, 0.74; *I*^2^ = 0%; *P* = 0.021) were significantly lower in the experimental group.

**Conclusion:**

Our systematic review and meta-analysis can prove the effectiveness and safety of ultrasound-guided acupotomy in the treatment of trigger finger, but this still needs to be verified by a clinical standard large sample test.

## Introduction

Trigger finger, also known as stenosing flexor tenosynovitis, is a common condition in the hand, characterized by catching, clicking, or locking of the fingers [1]. The etiology is versatile, including trauma, diabetes mellitus and inflammatory arthropathy. The most common pathology is flexor tendon nodules, stenosis of the pulley system, or a combination [2]. More women suffer from this disease than men. And the ring finger and thumb are the most prevalent trigger fingers [1].

Treatments for trigger finger include nonsteroidal anti-inflammatory agents (NSAIDs), massage, heat, ice, splinting, corticosteroid injections, extracorporeal shock wave therapy (ESWT), and surgery [3]. In recent years, acupotomy has been popularly used in clinical treatment in China [4]. Acupotomy is a modern type of acupuncture that uses a blade-needle combined with a flat surgical scalpel at its tip. Acupotomy can strip adhesions and release contractures of deep soft tissues [5]. And the mechanism of trigger finger treatment by acupotomy is similar to percutaneous A1 pulley release. The advantages of acupotomy for the trigger finger include a small wound size, short treatment time, high efficacy rate, and low recurrence rate.

However, the safety of acupotomy has often been questioned because it is a closed procedure that relies on sensations in the hands. Tendons, blood vessels and nerves may be injured during the treatment [6]. To solve this problem, ultrasound-guided acupotomy for the trigger finger has been widely used in recent years [7]. On the one hand, ultrasound images can clearly show the structural level of tissues, enabling us to accurately identify the location and type of lesions, and on the other hand, ultrasound images can accurately show the location of the acupotomy and its relationship with adjacent tissues with good accuracy and safety [8]. Studies have reported that ultrasound-guided acupotomy for the treatment of patients with cervical spondylosis [9], osteoarthritis of the knee [10] and tenosynovitis [11] improves clinical efficacy and safety. Pan et al. [12] reported that ultrasound-guided acupotomy for the trigger finger accurately revealed the width and location of the lesion while no complications occurred.

However, to date, there has been no systematic review or research project on ultrasound-guided acupotomy for trigger finger treatment. Therefore, this study analyzed and evaluated the clinical randomized controlled trials of ultrasound-guided acupotomy treatment by systematic evaluation and meta-analysis to assess its effectiveness and safety.

## Methods

### Search strategy

A systematic search was conducted in the following databases: the Cochrane Central Register of Controlled Trials (Cochrane Library), EMBASE, PubMed, China National Knowledge Infrastructure (CNKI), Wanfang Data, and Chinese Biomedical Literature Database (CBM) from the inception to January 2023. All searches were performed by a biomedical information specialist of the medical library, with an exhaustive set of search terms related to “ultrasound,” AND “acupotomy” OR “small needle knife” AND “trigger finger” OR “tenosynovitis” OR “tendonitis”. Chinese databases were also searched using the above Chinese search terms.

### Inclusion and exclusion criteria

We included studies if they met the following criteria: (1) randomized controlled trials (RCTs); (2) patients were diagnosed with trigger fingers regardless of gender, age, ethnicity, and nationality, and course of disease; (3) the experimental group was treated with ultrasound-guided acupotomy, and the control group was treated with traditional acupotomy, traditional operation or injection of medication.

We excluded the literature if: (1) repeated literature and summary descriptive literature; (2) animal experiments; (3) the language was not English or Chinese; (4) full texts were not available.

### Study selection

Two reviewers independently screened the title/abstract of each record by the inclusion criteria. And in cases of uncertainty, we retrieved the full text for further assessment. In the event of disagreement between the two researchers, a third researcher was consulted.

### Data extraction

Two researchers extracted data independently using a predetermined extraction table, and disagreements were resolved by consensus or by consulting a third researcher. We extracted the following data: (1) basic information; (2) participant’s baseline characteristics and inclusion/exclusion criteria; (3) details of intervention and control groups; and (4) outcomes (dichotomous data were number of events and total participants per group; continuous data were presented as mean ± standard deviation (SD), and total participants per group).

The primary outcome of this study was the overall clinical effectiveness of therapy. The efficacy evaluation criteria were as follows [13]. Excellent effective, no pain on the affected palm side, no pressure pain, free flexion and extension of fingers, no snapping; Effective, pain on the affected palm side was reduced after treatment, slight pain and mild snapping when moving; Ineffective, pain on the affected palm side was not reduced after treatment, strong pain when moving.

Secondary outcomes included (1) The Visual Analogue Scale (VAS) score (pain rating scales) [14]; (2) QuinneII classification (trigger finger severity) [15]; (3) incidence of complications; (4) recurrence rate.

## Quality assessment

The two authors conducted an independent assessment of the risk of bias using the Cochrane risk-of-bias tool to assess the methodological quality of the included studies [16]. The authors, organizations, journal titles, and findings of the included literature were unknown to the two authors. There were seven items in total, and each item was determined to be one of the following: “low risk of bias,” “unclear risk of bias,” and “high risk of bias.” If all seven items were assessed as having a low risk of bias, the study was rated as high quality. If one or more items were assessed as having a high or unclear risk of bias, the study was rated as low quality.

## Statistical analysis

Meta-analyses were performed using Review Manager 5.4 and Stata 15.1 software. For dichotomous data, we calculated the odds ratios (OR) with 95% confidence intervals (CI); for continuous data, we calculated weighted mean differences (WMD) with 95% CI. Statistical heterogeneity was assessed with the *I*^2^ statistic, with values > 50% indicating substantial heterogeneity. And we also considered sensitivity analyses where one study was excluded at a time. The Egger test was used to assess publication bias. Qualitative descriptive analysis was used if the heterogeneity of the included studies was large or the data could not be pooled. Two-sided *P* values < 0.05 were considered statistically significant.

## Result

### Result of study selection

**Fig. 1:**
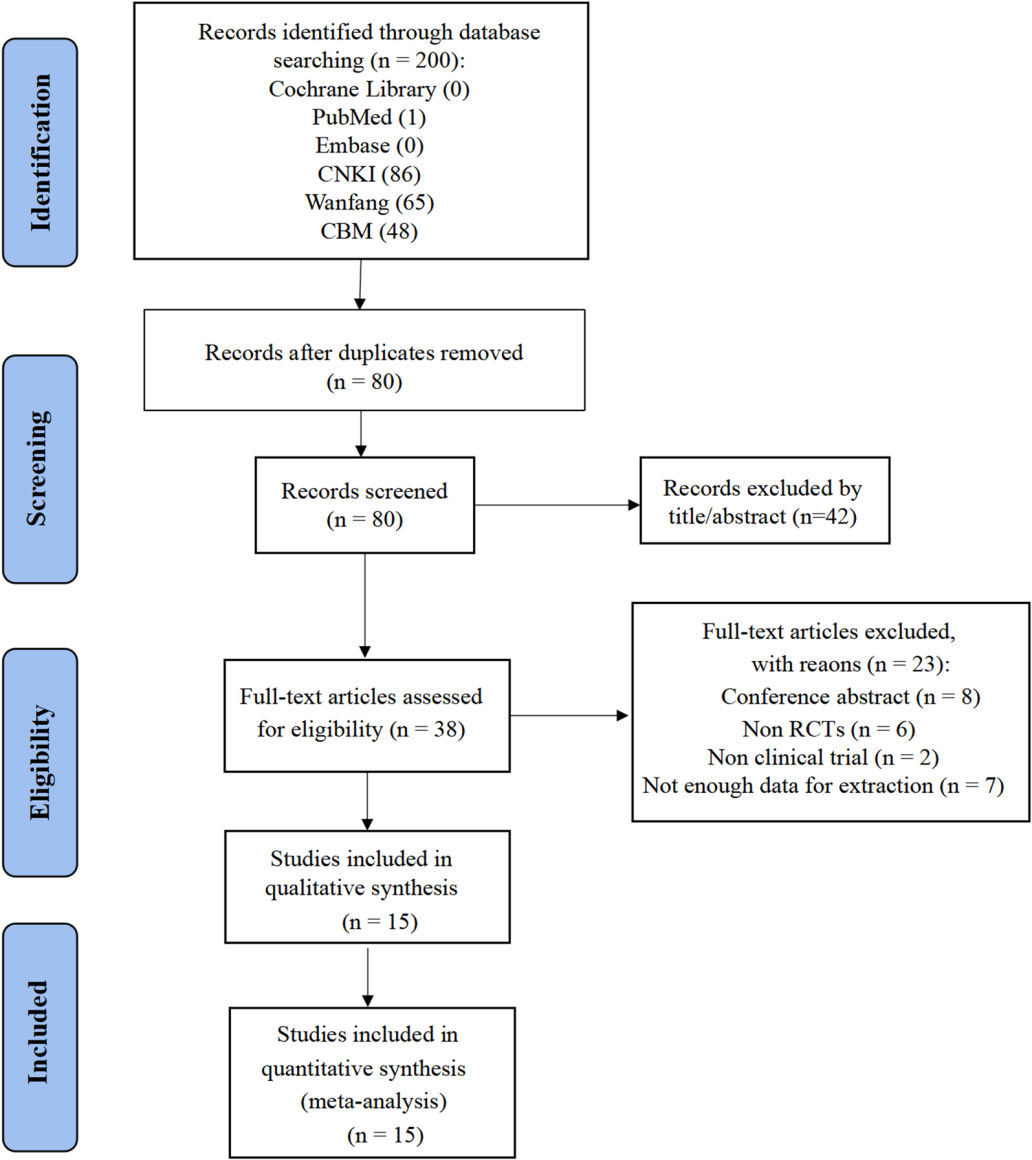
Flowchart of study selection method

A total of 200 records were retrieved, and after eliminating duplicates, the titles and abstracts of 80 records were screened. After a final screening, we included 15 studies [13, 17–30] with 988 patients. The screening process is detailed in Fig. 1.

### Study characteristics

**Fig. 2:**
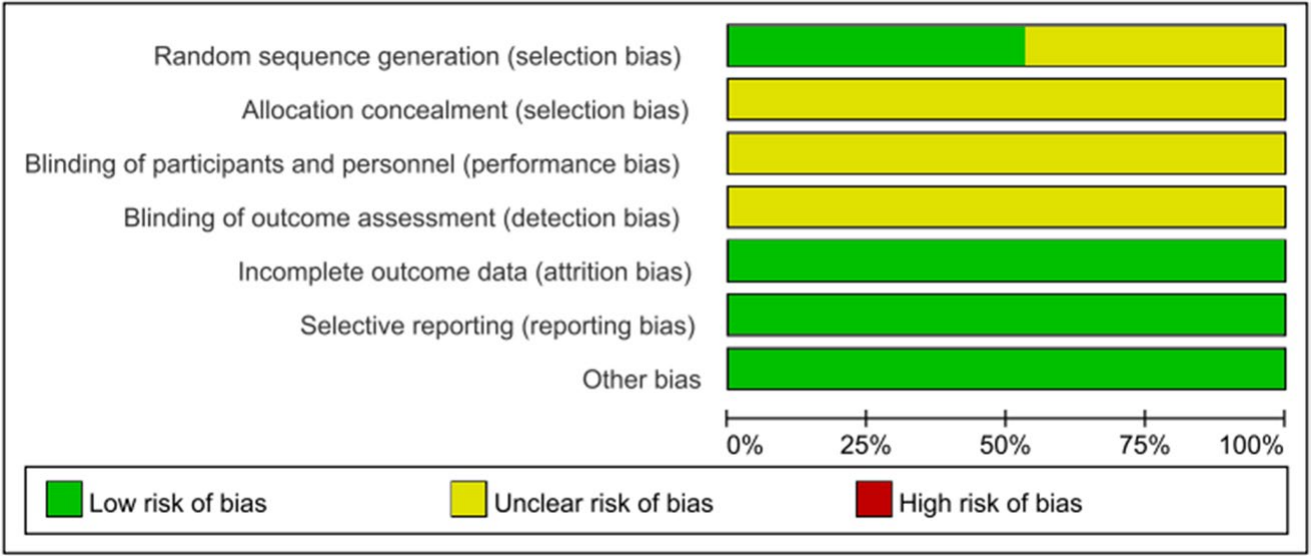
Risk of bias in the involved studies, assessed by using the Cochrane Collaboration’s risk-of-bias tool: high risk of bias (+); unclear risk of bias (?); and low risk of bias (−)

**Fig. 3:**
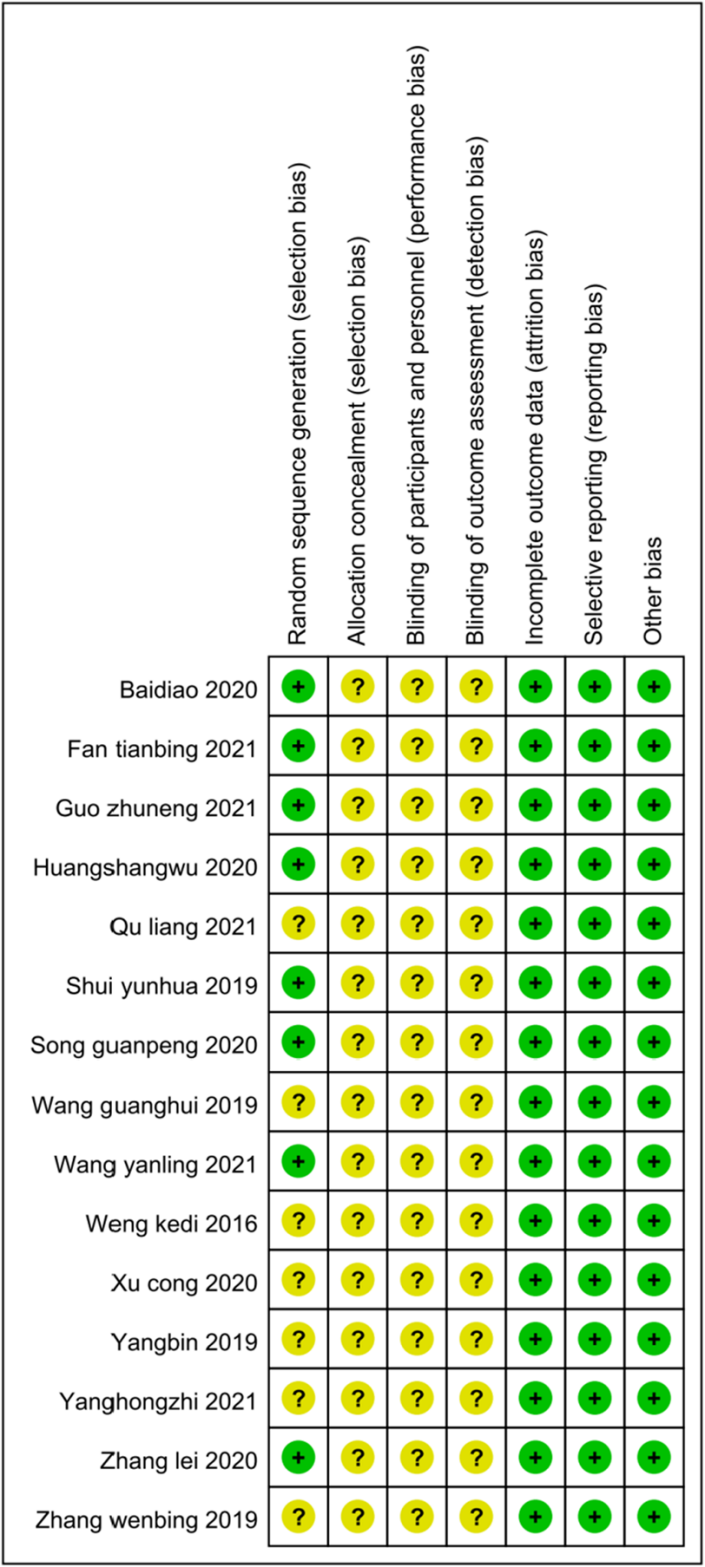
Graph of the risk of bias: percentage of all studies included

The main characteristics of the included trials are shown in Table 1. All of the RCTs were published between 2016 and January 2023. A total of 988 participants were aged between 18 and 75 years, and the duration of the diseases was between 3 and 42 months. The details of the quality assessment are shown in Figs. 2 and Fig. 3.

**Table 1:** The main characteristics of the included trials

Study	Sample size	Numbers of T/C (M/F)		Age (Year)		Duration (Months)		Intervention	
		Treatment	Control	Treatment	Control	Treatment	Control	Treatment	Control
Weng et al. [25]	60	30 (5/25)	30 (6/24)	46.7 ± 8.0	48.3 ± 8.2	3 ~ 18	3 ~ 18	Ultrasound-guided acupotomy	Acupotomy
Guo et al. [18]	40	20 (13/7)	20 (10/10)	52.8 ± 4.3	53.5 ± 4.6	25.2 ± 3.6	28.8 ± 6	Ultrasound-guided acupotomy	Surgery
Wang and Lin [24]	82	41 (14/27)	41 (13/28)	53.59 ± 6.31	53.61 ± 6.50	12.35 ± 5.76	12.48 ± 5.64	Ultrasound-guided acupotomy with corticosteroid injection	Ultrasound-guided corticosteroid injection
Song and Ge [22]	80	40 (23/17)	40 (21/19)	50.32 ± 2.01	51.32 ± 1.64	Not described	Not described	Ultrasound-guided acupotomy	Surgery
Wang et al. [23]	60	30 (3/27)	30 (4/26)	36.6 ± 2.1	37.1 ± 2	5.9 ± 2.5	6.0 ± 2.3	Ultrasound-guided acupotomy	Acupotomy
Zhang et al. [30]	74	37 (16/21)	37 (14/23)	48 ± 7	43 ± 5	4.4 ± 1.5	4.1 ± 1.7	Ultrasound-guided acupotomy with corticosteroid injection	Ultrasound-guided corticosteroid injection
Xu et al. [26]	112	68 (41/27)	44 (23/21)	54.32 ± 5.34	54.12 ± 5.31	36.84 ± 3.12	31.8 ± 3	Ultrasound-guided acupotomy	Surgery
Bai and Zhang [13]	30	15 (7/8)	15 (8/7)	67.2 ± 1.4	67.7 ± 1.31	18 ± 4.8	13.2 ± 3.6	Ultrasound-guided acupotomy	Corticosteroid injection
Yang [28]	80	40 (21/19)	40 (22/18)	38.67 ± 1.2	38.65 ± 1.21	20.04 ± 3.72	19.8 ± 14.52	Ultrasound-guided acupotomy	Corticosteroid injection
Zhang and Tang [29]	60	30 (8/22)	30 (9/21)	48 ± 7	43 ± 5	4.4 ± 1.5	4.1 ± 1.7	Ultrasound-guided acupotomy	Corticosteroid injection
Yang et al. [27]	60	30 (13/17)	30 (12/18)	45.11 ± 5.76	45.11 ± 5.76	Not described	Not described	Ultrasound-guided acupotomy	Surgery
Shui et al. [21]	90	60 (14/46)	30 (7/23)	46.22 ± 2.07	46.13 ± 2.03	11.17 ± 4.59	11.17 ± 4.59	Ultrasound-guided acupotomy	Acupotomy
Huang et al. [19]	60	30 (6/24)	30 (7/23)	59.62 ± 10.08	59.55 ± 9.27	6.76 ± 0.93	6.57 ± 1.20	Ultrasound-guided acupotomy with corticosteroid injection	Acupotomy with corticosteroid injection
Qu [20]	32	16 (1/15)	16 (2/14)	48.2 ± 3.1	48.5 ± 3.1	Not described	Not described	Ultrasound-guided acupotomy	Corticosteroid injection
Fan and Wang [17]	68	34 (22/12)	34 (20/14)	41.75 ± 4.29	42.63 ± 3.04	9.15 ± 2.43	9.86 ± 3.65	Ultrasound-guided acupotomy	Acupotomy

### Primary outcome

#### Overall clinical effectiveness

**Fig. 4:**
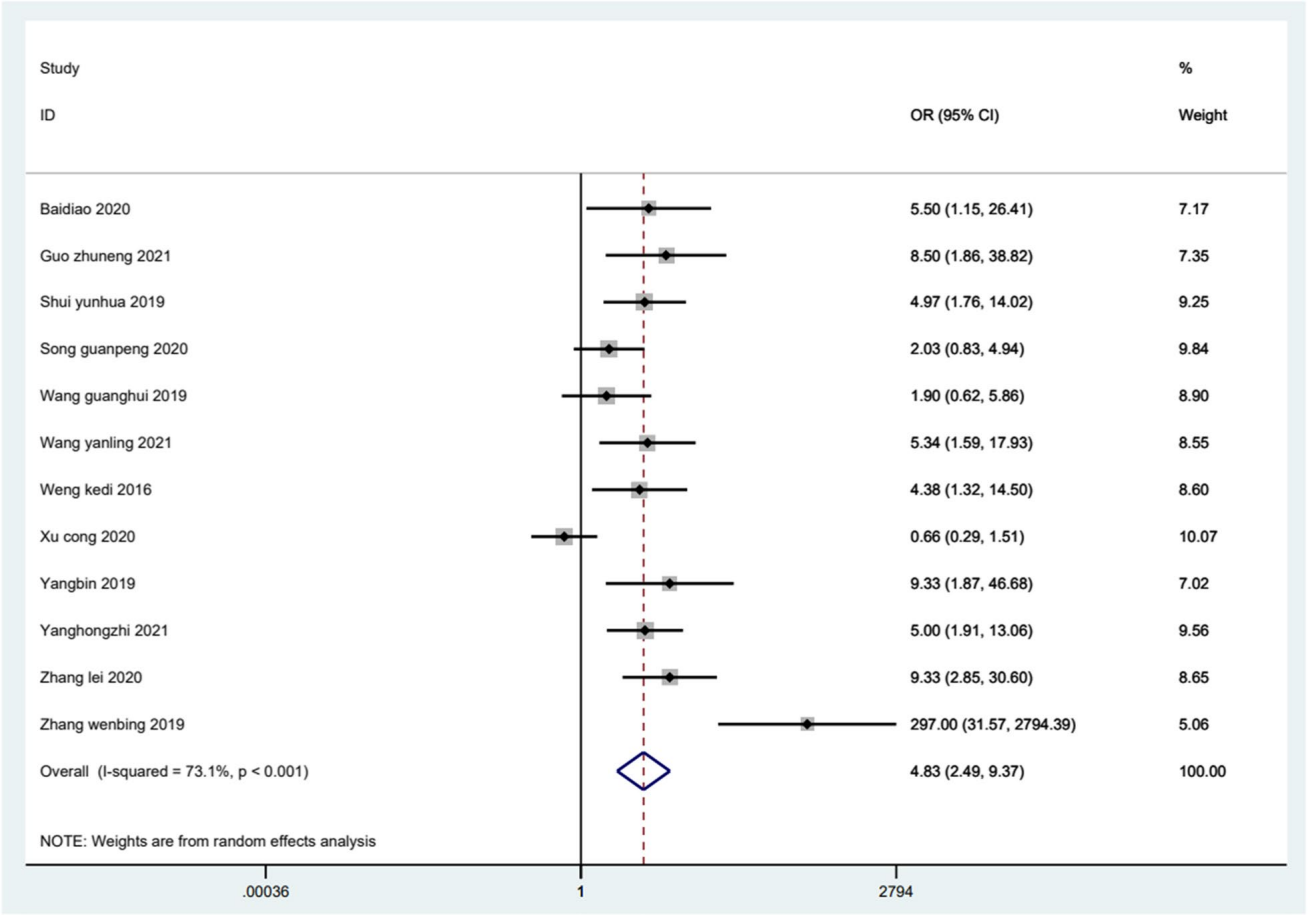
Meta-analysis on the overall clinical effectiveness of trigger finger treatment in the ultrasound-guided acupotomy group and control group

Fifteen studies have reported on the overall clinical effectiveness of ultrasound-guided acupotomy treatment. The results of our meta-analysis showed that the overall clinical effectiveness of trigger finger treatment was better in the ultrasound-guided acupotomy group than in the control group, and the difference was statistically significant (OR = 4.83; 95% CI 2.49–9.37; *I*^2 ^= 73.1%; *P *< 0.001) (Figure 4).

### Secondary outcomes

#### The VAS score

**Fig. 5:**
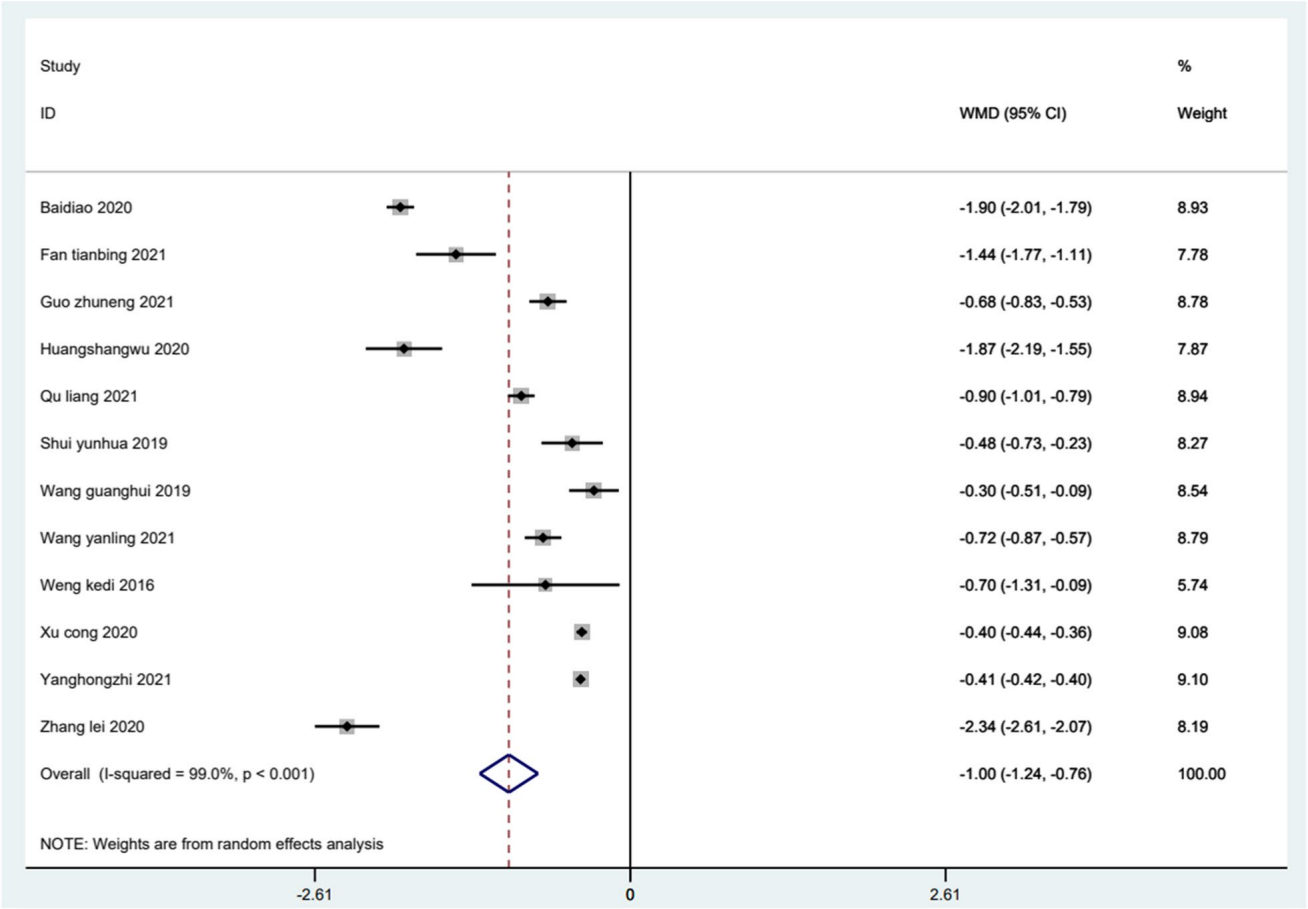
Meta-analysis on the Visual Analogue Scale (VAS) scores with trigger finger treatment in the ultrasound-guided acupotomy group and control group

Thirteen studies reviewed the VAS score with trigger finger. The results of the meta-analysis (Fig. 5) showed the VAS score of the ultrasound-guided acupotomy group was significantly lower than the control group, and the difference was statistically significant (WMD = − 1; 95% CI − 1.24, − 0.76; *I*^2^ = 99%; *P* < 0.001). It is suggested that the ultrasound-guided acupotomy group can reduce the pain of patients better than the control group.

#### QuinneII grading

**Fig. 6:**
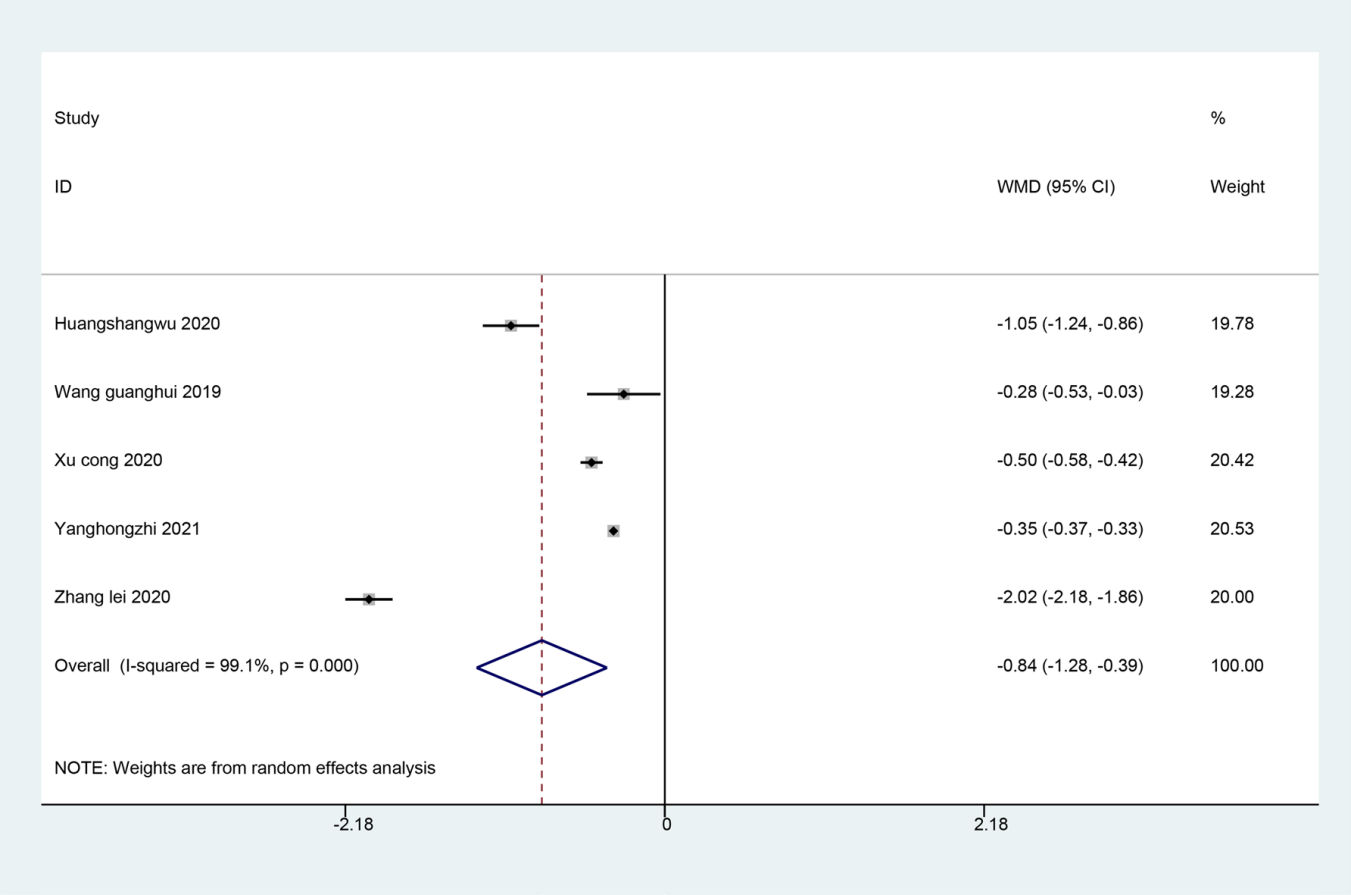
Meta-analysis on the QuinneII grading with trigger finger treatment in the ultrasound-guided acupotomy group and control group

Six studies reviewed the QuinneII grading with trigger finger. The results of the meta-analysis showed the QuinneII grading of ultrasound-guided acupotomy group was significantly lower than the control group, and the difference was statistically significant (WMD = − 0.84; 95% CI − 1.28, − 0.39; *I*^2^ = 99.1%, *P* < 0.001) (Fig. 6). It is suggested that the ultrasound-guided acupotomy group can reduce the QuinneII grading of patients better than the control group.

#### The incidence of complications

**Fig. 7:**
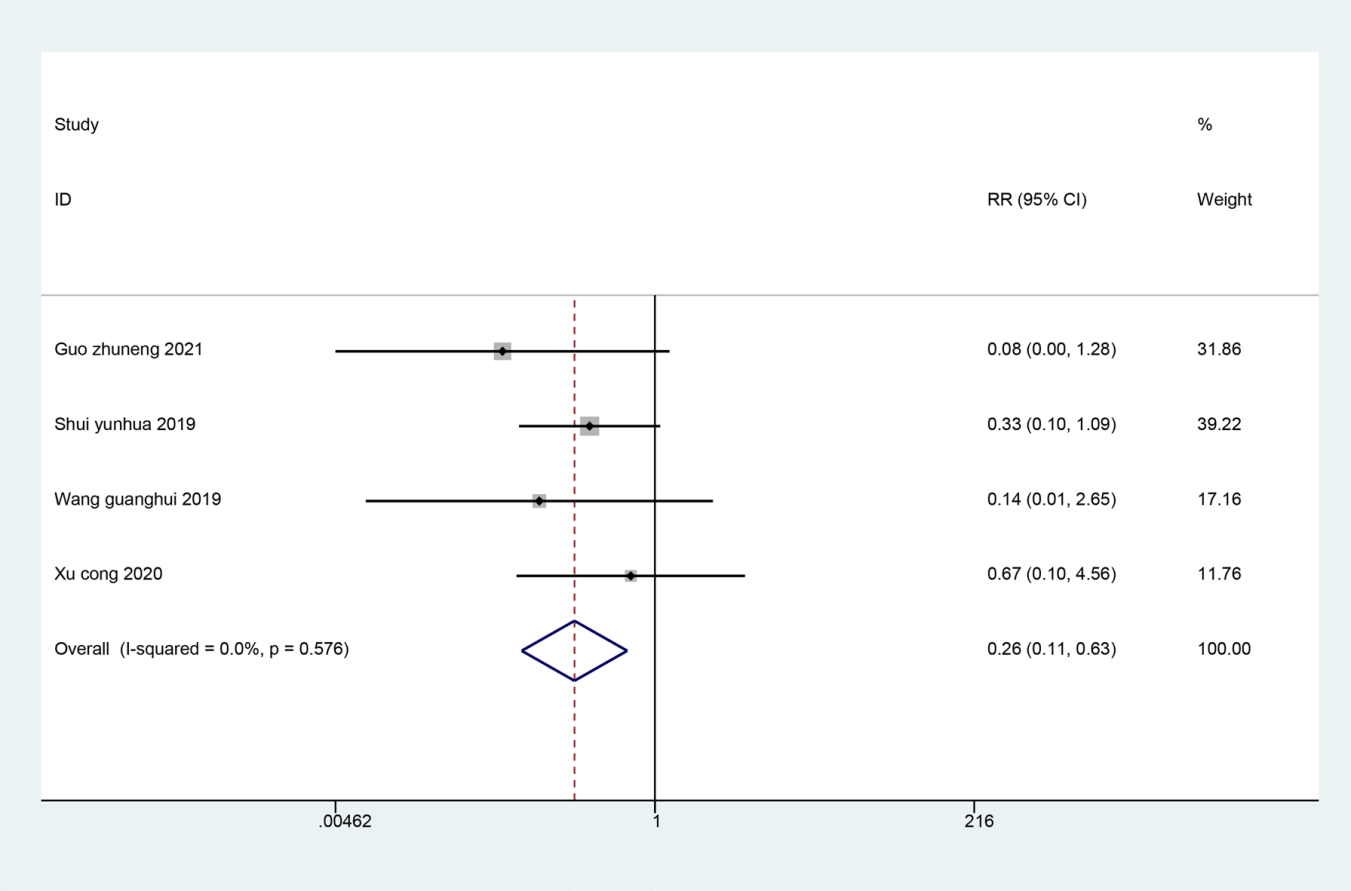
Meta-analysis on the incidence of complications after trigger finger treatment in the ultrasound-guided acupotomy group and control group

Four studies reviewed the incidence of complications after treatment. The results of the meta-analysis showed the incidence of complications in the ultrasound-guided acupotomy group was significantly lower than the control group, and the difference was statistically significant (RR = 0.26; 95% CI 0.11, 0.63; *I*^2^ = 0%, *P* = 0.003) (Fig. 7). It is suggested that the ultrasound-guided acupotomy group is safer than the control group.

#### The recurrence rate

**Fig. 8:**
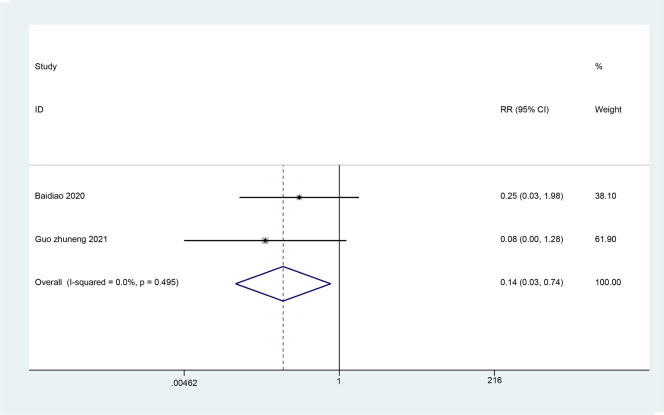
Meta-analysis on the recurrence rate of trigger finger treatment in the ultrasound-guided acupotomy group and control group

Two studies reviewed the recurrence rate. The results of the meta-analysis (Fig. 8) showed the recurrence rate of the ultrasound-guided acupotomy group was significantly lower than the control group, and the difference was statistically significant (RR = 0.14; 95% CI 0.03, 0.74; *I*^2^ = 0%; *P* = 0.021). It is suggested that the prognosis of the ultrasound-guided acupotomy group is better than the control group.

### Sensitivity analysis

**Fig. 9:**
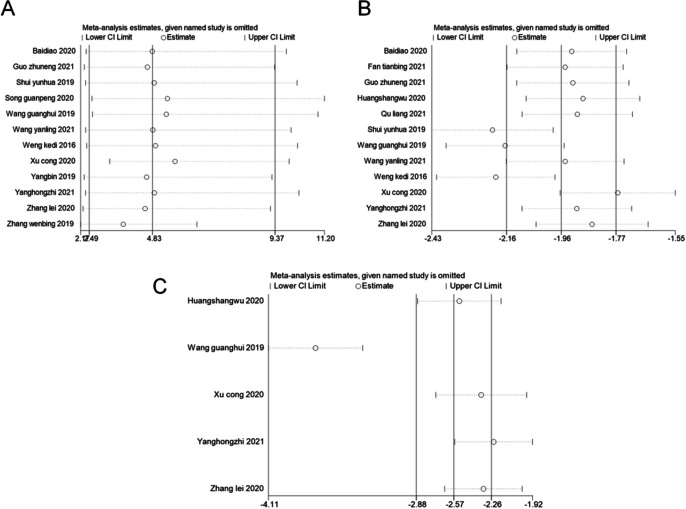
Sensitivity analyses of overall clinical effectiveness (**A**), VAS score (**B**) and QuinneII classification (**C**) in patients with Trigger finger

We performed sensitivity analyses using a *metaninf* command in the STATA software for overall clinical effectiveness, the VAS score and the QuinneII grading. And the results were not significantly different from those of the primary analysis (Fig. 9A–C).

### Publication bias

**Fig. 10:**
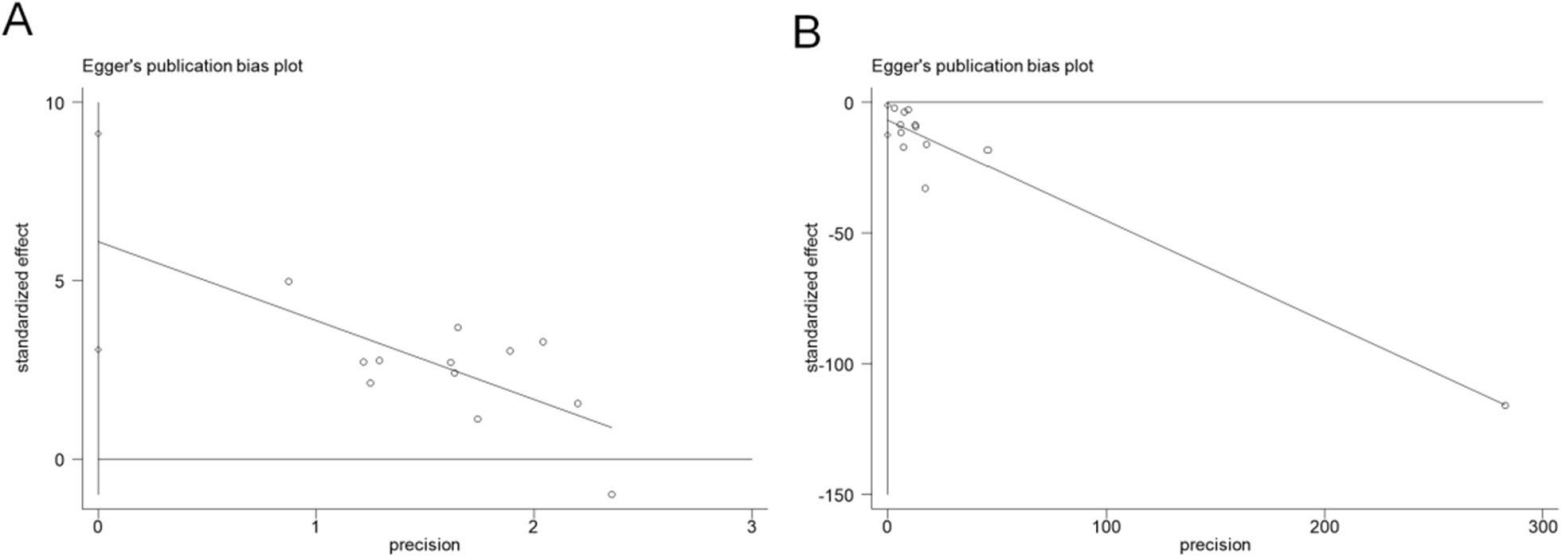
Publication bias test of overall clinical effectiveness (**A**) and VAS score (**B**) in patients with Trigger finger

We used the *metabias6* command in the STATA software to test the publication bias. The results revealed the existence of publication bias in the meta-analysis of overall clinical effectiveness (Fig. 10A; *P* >|*t*|= 0.001) and VAS scores (Fig. 10B; *P* >|*t*|= 0.022).

## Discussion

Our study systematically evaluated the effect and safety of ultrasound-guided acupotomy on trigger finger. The study results showed that ultrasound-guided acupotomy had better effects than the control group. There may be two reasons for this. First, ultrasound-guided acupotomy can accurately determine the location of the percutaneous A1 pulley, directly to the diseased region. Second, ultrasonic real-time dynamic monitoring can avoid iatrogenic injury to important nerves and blood vessels, thereby reducing the incidence of adverse events, reducing swelling and fluid and other complications [31].

And the study also showed that the ultrasound-guided acupotomy group can reduce the pain and the QuinneII grading of patients better than the control group. The pain of the trigger finger before and after treatments was determined with a VAS score [32]. And the QuinneII grading was a clinical grading of finger function from 0 to 4 grades [15]. As ultrasound can show the thickening lesion of A1 pulley clearly and dynamically observe the sliding of the tendon in the sheath, operators can use acupotomy to release A1 pulley accurately and thoroughly, reducing secondary damage and pain, conducive to early finger activity function exercise to avoid or reduce the adhesion recurred again.

In addition, the study showed that the incidence of complications and recurrence rate in the ultrasound-guided acupotomy group was significantly lower than the control group, which suggested that the ultrasound-guided acupotomy group was safer than the control group. Because under the guidance of ultrasound visualization, the operators could see the blood vessels and nerves clearly and avoid damaging them. Yang et al. showed that ultrasound-guided acupotomy release was safer than the needle, and no vascular nerve injury or A2 pulley injury occurred. It was confirmed that ultrasound-guided percutaneous A1 pulley release by acupotomy is a safe and effective method [33].

However, this study may have the following limitations. First, the small number and the low quality of the studies included in this review may have affected our results. Second, the control group was treated with acupuncture, surgery, and corticosteroid injections, which may have been biased. And only a few articles have studied adverse reactions, so the safety of ultrasound-guided acupotomy needs to be further observed.

As a new therapeutic technique, it is still being explored, and there are many aspects that need to be further improved. According to the data and results of this study, we find that sample size should be increased in future studies, the rigor of trial design should be improved, and multi-center RCT should be used to provide more objective clinical evidence.

## Conclusion

Our systematic review and meta-analysis can prove the effectiveness and safety of ultrasound-guided acupotomy in the treatment of trigger finger, but this still needs to be verified by a clinical standard large sample test.

## Data Availability

The data used to support the findings of this study are available from the corresponding author upon request.
